# Mindfulness-based real-time fMRI neurofeedback: a randomized controlled trial to optimize dosing for depressed adolescents

**DOI:** 10.1186/s12888-023-05223-8

**Published:** 2023-10-17

**Authors:** Paul A. Bloom, David Pagliaccio, Jiahe Zhang, Clemens C. C. Bauer, Mia Kyler, Keara D. Greene, Isaac Treves, Francesca Morfini, Katherine Durham, Rachel Cherner, Zia Bajwa, Emma Wool, Valur Olafsson, Ray F. Lee, Fred Bidmead, Jonathan Cardona, Jaclyn S. Kirshenbaum, Satrajit Ghosh, Oliver Hinds, Paul Wighton, Hanga Galfalvy, H. Blair Simpson, Susan Whitfield-Gabrieli, Randy P. Auerbach

**Affiliations:** 1 Department of Psychiatry, Columbia University, New York, NY USA; 2 Department of Psychology, Northeastern University, Boston, MA USA; 3 Department of Brain and Cognitive Sciences, Massachusetts Institute of Technology, Cambridge, MA USA; 4 Northeastern University Biomedical Imaging Center, Boston, MA USA; 5 Zuckerman Mind Brain and Behavior Institute, Columbia University, New York, NY USA; 6 Harvard Medical School, Boston, MA USA; 7 Orchard Scientific, Yucca Valley, CA USA; 8 Athinoula A. Martinos Center for Biomedical Imaging, Massachusetts General Hospital, Charlestown, MA USA

**Keywords:** Adolescence, MDD, Real-time fMRI neurofeedback, Mindfulness, DMN, FPN

## Abstract

**Background:**

Adolescence is characterized by a heightened vulnerability for Major Depressive Disorder (MDD) onset, and currently, treatments are only effective for roughly half of adolescents with MDD. Accordingly, novel interventions are urgently needed. This study aims to establish mindfulness-based real-time fMRI neurofeedback (mbNF) as a non-invasive approach to downregulate the default mode network (DMN) in order to decrease ruminatory processes and depressive symptoms.

**Methods:**

Adolescents (*N* = 90) with a current diagnosis of MDD ages 13–18-years-old will be randomized in a parallel group, two-arm, superiority trial to receive either 15 or 30 min of mbNF with a 1:1 allocation ratio. Real-time neurofeedback based on activation of the frontoparietal network (FPN) relative to the DMN will be displayed to participants via the movement of a ball on a computer screen while participants practice mindfulness in the scanner. We hypothesize that within-DMN (medial prefrontal cortex [mPFC] with posterior cingulate cortex [PCC]) functional connectivity will be reduced following mbNF *(Aim 1: Target Engagement).* Additionally, we hypothesize that participants in the 30-min mbNF condition will show greater reductions in within-DMN functional connectivity *(Aim 2: Dosing Impact on Target Engagement).* Aim 1 will analyze data from all participants as a single-group, and Aim 2 will leverage the randomized assignment to analyze data as a parallel-group trial. Secondary analyses will probe changes in depressive symptoms and rumination.

**Discussion:**

Results of this study will determine whether mbNF reduces functional connectivity within the DMN among adolescents with MDD, and critically, will identify the optimal dosing with respect to DMN modulation as well as reduction in depressive symptoms and rumination.

**Trial Registration:**

This study has been registered with clinicaltrials.gov, most recently updated on July 6, 2023 (trial identifier: NCT05617495).

## Introduction

Depression is a leading cause of disability worldwide [1]. Among adolescents, estimated rates of major depressive disorder (MDD) are as high as 15–20% in the United States [2, 3], and prevalence has been increasing over the last several decades [4, 5]. Unfortunately, many adolescents with MDD do not receive treatment [6], and even established psychological and pharmacological treatments are only effective for approximately half of patients [7–9]. Thus, novel approaches for treatment are needed.

A potentially impactful target for treatment may be rumination, or the perseveration of negative self-referential thoughts, memories, and one’s own negative mood [10, 11]. Rumination is a common component of MDD [12], as well as other affective disorders [13], and a preponderance of research has shown that a ruminative response style increases the severity and duration of depression [14, 15]. Moreover, prior work indicates that patients with MDD reporting greater ruminative tendencies are less likely to respond to existing treatments and relapse more frequently [16, 17]. Therefore, interventions targeting rumination may be a promising avenue for adolescent depression treatment.

Recent neuroimaging research has observed increased activation during rumination compared to control conditions within the default mode network (DMN), which has core hubs in cortical midline regions (medial prefrontal cortex [mPFC], posterior cingulate cortex [PCC]) as well as the medial temporal lobe (MTL) and angular gyrus [18–21]. The DMN is generally activated during self-relevant processing, including mind-wandering, and deactivated during focus on the external environment, typically showing negative functional correlations with task-positive networks [22, 23]. Recent work in healthy adults suggests that rumination is associated with increased connectivity between core midline hubs of the DMN [24], though findings have differed somewhat across studies and tasks [25–27]. Further, rumination is associated with functional connectivity between the DMN and both the salience network (SN) and the frontoparietal network (FPN; also sometimes referred to as the frontoparietal control network or central executive network), which is associated with executive function and focused attention [28–30]. Taken together, brain imaging evidence highlights that rumination may involve brain network activations and connectivity related to self-referential, repetitive thinking, and a lack of top-down attentional control.

Mindfulness-based treatments, which train attentional focus on the present moment, are one approach to treating depression through targeting reductions in rumination symptoms [31, 32]. In particular, a goal of many mindfulness treatments is to enable individuals to become aware of when negative thoughts occur and to notice them without judgment or perseveration [33]. In addition to stand-alone mindfulness-based treatments, mindfulness skills are a core component of several types of behavioral (e.g., Dialectical Behavior Therapy) [34] and integrative psychotherapies for adolescents.

Although mindfulness approaches show comparable treatment outcomes relative to other first-line approaches (e.g., cognitive behavior therapy, antidepressant medication) [35–37], recent work suggests that there may be opportunities to optimize the delivery, particularly in the context of MDD among youth [38]. One reason for varied treatment effects may be that mindfulness-based treatments differ in delivery (e.g., clinician-guided vs. mobile app-based) and content [39]. Additionally, symptoms experienced during a depressive episode (e.g., heightened self-criticism, inattention) may exacerbate ruminative thoughts and reduce attentional control, which may impede effective mindfulness practices [40]. Studies have shown that practice quality matters [41], and those individuals who experience greatest mindfulness in the moment of practice show the most enduring changes in their daily life [42]. Accordingly, timely feedback during mindfulness may afford a promising avenue to improve mindfulness skill acquisition and result in positive clinical outcomes among depressed adolescents.

An unexplored potential avenue for enhancing mindfulness interventions for adolescent MDD is real-time neurofeedback, a procedure in which individuals receive moment-by-moment visual or auditory feedback based on their brain signals (e.g. electroencephalography, fMRI, magnetoencephalography) [43, 44]. As a treatment, neurofeedback aims to help individuals build awareness and control of mental processes by guiding them towards a “target” brain state [45]. To date, there is encouraging progress applying real-time neurofeedback as a treatment for depression [46–48], but relatively few studies have used real-time neurofeedback to augment mindfulness. Within mindfulness-based treatments, neurofeedback may help individuals recognize when they are focused on the present moment versus ruminating about negative experiences [49]. Testing augmentation of mindfulness practice with real-time neurofeedback will be particularly important for adolescents with MDD given the high need for novel and personalized treatments.

A further critical gap is that few real-time neurofeedback studies have tested network-based targets, in particular the DMN and FPN, for downregulating rumination symptoms or treating MDD [43, 50, 51]. Most real-time fMRI studies for MDD have trained individuals to upregulate amygdala signal or other limbic regions during positive autobiographical recall [52–55]. Prior work indicates that healthy individuals are able to upregulate or downregulate DMN activity via real-time fMRI neurofeedback [49, 56–58], but the downstream impacts on rumination and depression symptoms remain unknown. Preliminary results from a recent proof-of-concept trial suggest reduced rumination among depressed adults following real-time neurofeedback targeting PCC connectivity with the right temporoparietal junction [59]. However, additional research is needed to evaluate the efficacy of DMN-based real-time neurofeedback, especially for adolescent MDD. Given recent findings that transcranial magnetic stimulation treatments decrease depression symptoms through decreasing functional connectivity between dorsolateral prefrontal cortex (a core FPN region) and the subgenual anterior cingulate cortex (sgACC), further investigation of FPN and DMN real-time neurofeedback targets are critical [60–63]. Addressing this gap, our recent pilot work demonstrated decreased functional connectivity within the DMN among adolescents with a history of affective disorders following a single 15-min session of real-time fMRI neurofeedback [64].

Building on this pilot research, the current project aims will test real-time fMRI neurofeedback combined with mindfulness practice and, crucially, whether such an intervention is effective for adolescents with MDD. Specifically, the current study will clarify whether DMN modulation is a mechanism of action for improving rumination and depressive symptoms in depressed adolescents and test different dosing lengths. Many real-time neurofeedback treatment studies use multiple feedback sessions [65], though some studies have shown improvement on clinical outcomes after only 1–2 sessions [66] and time course changes may vary [67]. As MRI scans are costly and are potentially burdensome, developing briefer real-time neurofeedback interventions will help increase the accessibility of treatment. Therefore, we seek to establish the optimal duration of a session of real-time neurofeedback.

In summary, the current randomized clinical trial (RCT) (ClinicalTrials.gov Identifier #NCT05617495; R61MH132072) will enroll depressed adolescents ages 13–18-years-old. Participants will be randomized to either a 15-min or 30-min dose of mindfulness-based neurofeedback (mbNF) and clinical assessments will be completed over a one-month follow-up. The following hypotheses will be tested. First, a single session of mbNF will lead to reductions in within-DMN functional connectivity *(Aim 1: Target Engagement)*. Second, a 30-min session will lead to greater reduction in within-DMN functional connectivity compared to the 15-min session *(Aim 2: Dosing Impact on Target Engagement)*. As all participants will receive an active mbNF intervention, the current study is regarded as open-label. Aim 1 analyses will treat the study as a single-group trial, and analyses for aim 2 will leverage randomization to treat the study as a parallel-group superiority trial.

## Methods

### Participants

Eligible participants will be 13–18-year-old adolescents with a current diagnosis of MDD, assessed via the Kiddie-Schedule for Affective Disorders and Schizophrenia (K-SADS) [68]. Participants will be fluent in English, capable of giving assent (or consent for 18-year-olds), and report ≥ 3 on the Tanner Stage puberty assessment to minimize neuroendocrine variability in the sample [69]. In addition, participants must have access to a smartphone to complete the Ecological Momentary Assessment (EMA), though access to a smartphone will not be required for inclusion for other study procedures. Participants will be excluded if they have a lifetime history of primary psychotic disorders, bipolar disorders, oppositional defiant disorder, conduct disorder, post-traumatic stress disorder, eating disorders, or a developmental disorder. Participants also will be excluded if they have a moderate or severe substance use disorder in the past 6 months, use of psychotropic medications (except for antidepressant medication), a history of seizure or other neurological disorder, a Full-scale Intelligence Quotient (FSIQ-2) scaled score of < 80 using the Wechsler Abbreviated Scale of Intelligence-II (WASI-II) [70], or any MRI contraindications. Any participant who has active suicidal ideation with some intention of acting in the past week, as assessed by the Columbia-Suicide Severity Rating Scale (C-SSRS) [71], will be excluded (see suicide risk management plan at https://github.com/pab2163/mindful_brain_project/tree/main/materials). Suicide risk may also be identified via the K-SADS or Children’s Depression Rating Scale-Revised. If suicide risk is detected at any session, a licensed clinician will complete a safety evaluation and when necessary, bridge participants with appropriate clinical services.

Participants will be recruited from Columbia University (CU) and Northeastern University (NEU) with a goal to enroll 110 participants equally divided by sex assigned at birth, with 90 fully completing the study (45 per site). Study advertisements will be placed in various locations in the greater New York/New Jersey and Boston areas, including Columbia University Irving Medical Center (CUIMC), New York State Psychiatric Institute (NYSPI), and NEU. Advertisements also will be posted online on social media (e.g., Facebook, Instagram). Individuals also will be recruited through clinician referrals.

### Screening

Screening, baseline, and follow-up assessments will all be conducted via a HIPAA-compliant video conferencing platform (Table 1). Study staff will join these videoconferences from a private area to ensure participant privacy. Adolescents under 18-years-old will provide informed written assent and a parent or legal guardian will provide permission for the adolescent to participate. Adolescents who are 18 years old will provide informed written consent. After the assent/consent process, inclusion/exclusion criteria will be confirmed via screening instruments (C-SSRS, metal screen, WASI-II, Tanner Scale, Service Use Questionnaire, K-SADS). All adolescents will complete the K-SADS, and when possible, the K-SADS also will be administered to caregivers about their child’s psychiatric history (for participants under age 18). If caregivers are unreachable at the scheduled time for K-SADS interviews, 3 attempts will be made (text, email, and/or phone call), after which eligibility will be determined using only the K-SADS completed with the adolescent. Participants will be administered a service-use interview to assess lifetime treatment utilization (e.g., medication, therapy) and history of mindfulness practice. After all screening procedures have been completed, study staff will review and confirm eligibility. Participants will be remunerated $50 (USD) for completing the screening assessment.

**Table 1:** Schedule of screening measures and assessments for clinical instruments

**Measurement**	Visit 0 (Screening)	Visit 1 (Baseline)	Visit 2 (Start of Visit)	Visit 2 (Post-Mindfulness)	Visit 2 (Post-mbNF)	Visit 3 (Follow-Up)
**Screening & Baseline Measures**						
*Wechsler Abbreviated Scale of Intelligence (WASI-II)*	X					
*Service Use Interview*	X					X
*Kiddie Schedule for Affective Disorders and Schizophrenia (K-SADS)*	X					
*Columbia Suicide Severity Rating Scale (C-SSRS)*	X		X			X
*Tanner Scale*	X					
*Demographics Interview*		X				
*Chapman Handedness Inventory*		X				
**Clinical Interviews**						
*Children's Depression Rating Scale-Revised (CDRS-R)*		X				X
**Anxiety- and Depression-Related Self-Report Scales**						
*Mood and Feeling Questionnaire (MFQ)*		X			X	X
*Revised Child Anxiety and Depression Scale (RCADS)*		X			X	X
*Perceived Stress Scale (PSS)*		X	X		X	X
*Positive and Negative Affect Schedule-Short Form (PANAS-SF)*		X			X	X
**Rumination-Related Self-Report Scales**						
*Ruminative Response Scale (RRS)*		X			X	X
*Perseverative Thinking Questionnaire (PTQ)*		X			X	X
**Mindfulness-Related Self-Report Scales**						
*Five-Facet Mindfulness Questionnaire (FFMQ)*		X			X	X
*State Mindfulness Scale (SMS)*		X	X		X	X
*Mind-Wandering Questionnaire (MWQ)*		X			X	X
**Debriefing and Brief Self-Report Scales**						
*MRI Protocol Debrief Questionnaire*					X	
*Brief Fatigue, Stress, and State Mindfulness Assessment*			X	X	X	

### Baseline assessment

For eligible participants, baseline assessments will be conducted within approximately one week after screening. At baseline, participants will complete a series of self-report questionnaires including the Mood and Feelings Questionnaire (MFQ) [72], Revised Children’s Anxiety and Depression Scale (RCADS) [73], and Perceived Stress Scale (PSS) [74]. We also will collect self-report measures of rumination (Ruminative Response Scale [RRS] [11], Perseverative Thinking Questionnaire [PTQ] [75]), mindfulness (Five-Facet Mindfulness Questionnaire [FFMQ] [76], State Mindfulness Scale [SMS] [77]), mind-wandering (Mind-Wandering Questionnaire [MWQ] [78]), affect (Positive and Negative Affect Schedule-Short Form [PANAS-SF] [79], and handedness (Chapman Handedness Inventory [80]). Adolescents also will be administered the Children’s Depression Rating Scale-Revised (CDRS-R) [81] to ascertain depression symptom severity, and complete a demographic interview. During the baseline assessment, participants will install the Metricwire smartphone app onto their personal smartphone. Participants will be remunerated $50 (USD) for completing the baseline assessment.

### Ecological Momentary Assessment (EMA) surveys

**Fig. 1:**
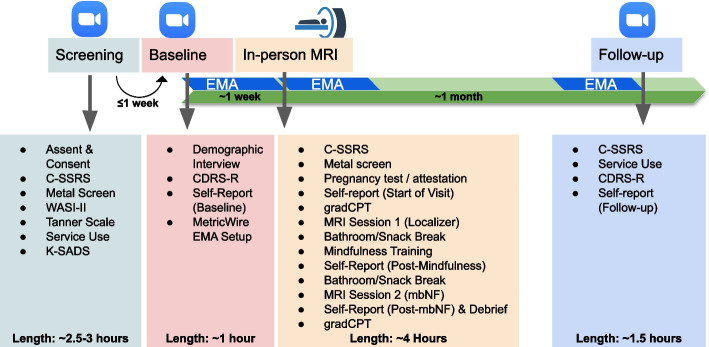
Protocol Overview. Screening and baseline assessment (videocall) will be followed by the in-person MRI visit approximately 1 week after baseline. Follow-up assessments will be approximately 1 month after the MRI visit. Ecological Momentary Assessment (EMA) prompts will be delivered during the week after the baseline assessment and MRI visit, as well as the week leading up to the follow-up assessment. WASI-II = Wechsler Abbreviated Scale of Intelligence-II, K-SADS = Kiddie-Schedule for Affective Disorders and Schizophrenia, CDRS-R = Children’s Depression Rating Scale-Revised, C-SSRS = Columbia-Suicide Severity Rating Scale, mbNF = mindfulness-based real-time fMRI neurofeedback, gradCPT = gradual onset continuous performance task. For the current protocol, visits refer to study interactions on separate days (Screening visit, baseline visit, MRI visit, and 1-month follow-up visit), while MRI sessions refer to continuous windows of time within visits when the participant is in the scanner (MRI Session 1, MRI Session 2). A free stock image from Vecteezy.com was used in this figure

Participants will complete 3, 1-week blocks of EMA: (1) following the baseline clinical characterization, (2) following the MRI visit, and (3) 1 week prior to the 1-month follow-up assessment (See Fig. 1). EMA will be completed via Metricwire [82], a HIPAA-compliant app downloaded to their personal smartphone which will deliver four surveys per day (7am, 2 pm, 5 pm, 7 pm), each lasting ~ 2–3 min. Surveys will include prompts about rumination, stress, and mindfulness, as well as items adapted from the Patient Health Questionnaire-2 (PHQ-2) and General Anxiety Disorder 2-Item (GAD-2) for assessing depression and anxiety symptoms, respectively (see https://github.com/pab2163/mindful_brain_project/tree/main/materials). Each item will be rated on a scale from 0 (*not at all*) to 100 (*all the time*), and item order will be counterbalanced across assessments. A control item *“Please identify the center of the response bar, as this will help us score your responses”* will be used to assess response quality once per survey. Participants will be remunerated $1 per EMA survey (up to $84 total).

### Randomization

Participants will be randomly assigned to one of two dose conditions on the day of the MRI visit: 15 min or 30 min of mbNF. As the dose conditions are the duration of mbNF, participants cannot be blinded, nor will the study staff administering the scan visit. Randomization will be stratified by site, sex assigned at birth, prior mindfulness experience (3 + mindfulness sessions), and current treatment (i.e., Yes vs. No). Participants will be randomized within strata, and it is possible that sample sizes will differ between strata. Randomization is implemented in REDCap. Staff conducting the follow-up clinical interview will remain blind to the dosing assignment.

### MRI visit

Approximately one week after the baseline assessment, participants will complete an in-person visit to the MRI center, where the mbNF will be administered. Visits will take approximately 4 h. At the start of the visit, participants will complete an MRI safety screener and the C-SSRS screener. Per institutional policies, current pregnancy is contraindicated for MRI; participants will either attest (NEU) or complete urine screening (CU) to confirm pregnancy status. Before completing MRI Session 1 (Functional Localizer), participants will complete a 4-min gradual onset continuous performance task (gradCPT) to assess attentional control [83] and the State Mindfulness Scale (SMS) and Perceived Stress Scale (PSS), as well as a brief assessment of fatigue.

Next, the participant will complete a 30–40-min MRI Session 1 (Functional Localizer). Participants will then exit the scanner, have an opportunity for a snack and bathroom break, and will complete mindfulness training (~ 45 min). During the mindfulness training, participants’ fMRI data from MRI Session 1 will be used to create personalized DMN & FPN masks which will be used to deliver personalized real-time neurofeedback. Participants next complete a second brief assessment of state mindfulness, stress, and fatigue. Participants then will complete MRI Session 2 (mbNF), which will last approximately 75–90 min. Afterwards, participants will complete an MRI debriefing questionnaire (which will include questions on participants’ experiences with the feedback and strategies used), as well as the gradCPT a second time, and the MFQ, RCADS, PSS, RSS, PTQ, FFMQ, SMS, MWQ, PANAS-SF, and brief fatigue assessment. All questionnaires at the MRI visit will be completed on REDCap using an iPad or laptop. Participants will be remunerated $150 for the MRI scanning, $50 for the mindfulness training, and $50 for transportation.

### MRI session 1 (functional localizer)

**Fig. 2:**
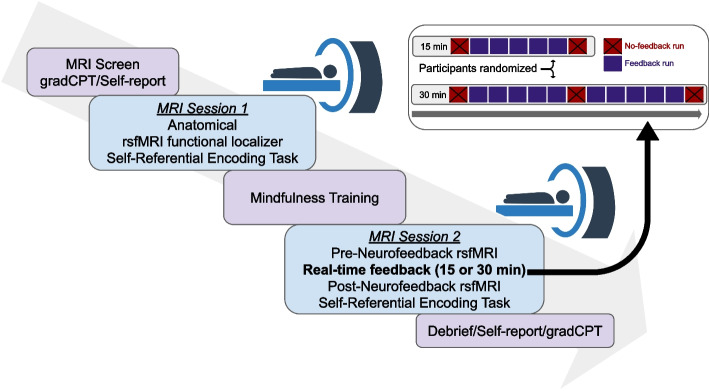
MRI Visit Overview. Participants will be randomized to receive either a 15-min or 30-min dose of mbNF at MRI session 2. In the upper right hand corner, shaded rectangles indicate 3-min fMRI runs with (blue) versus without (red, with X) real-time neurofeedback. gradCPT = gradual onset continuous performance task, rsfMRI = resting-state functional magnetic resonance imaging. A free stock image from Vecteezy.com was used in this figure

The rsfMRI data acquired at MRI Session 1 will be used for functional localization of personalized DMN and FPN maps (Fig. 2). At both sites, MRI data will be acquired on a Siemens Prisma scanner with a 64-channel head coil. A T1-weighted MPRAGE anatomical scan [1 mm isotropic voxel size, 176 slices, field-of-view (FOV) = 256 × 256 × 176 mm, repetition time (TR) = 2530 ms, echo time (TE) = 1.92 ms, flip angle (FA) = 7°] will be acquired. During collection of the MPRAGE, participants will watch a movie without sound, narrative, or scene cuts (Inscapes) [84] to help acclimate to the scanner environment. Two images with opposing phase encoding direction will be acquired to generate a fieldmap offline, followed by two 5-min runs of resting state fMRI (rsfMRI) (multiband acceleration factor = 4, 2 mm isotropic voxel size, 72 slices, FOV = 256 × 256 × 144 mm, TR = 1200 ms, TE = 30 ms, FA = 61°, 250 total volumes, phase-encoding *P* >  > A). For the current protocol, a “run” refers to a single period of MRI data acquisition (and simultaneous behavior) in which data are acquired without pause using a single sequence. Full MRI protocols with all parameters can be found at https://github.com/pab2163/mindful_brain_project/tree/main/materials. RsfMRI will be collected using a standard Siemens SMS echo-planar imaging (EPI) sequence configured with a VSend reconstruction functor [45, 85] that allows the EPI volumes to be sent in real time to a laptop in the MRI control room via ethernet connection [86]. During rsfMRI, participants will be instructed to relax and keep their eyes open while a fixation cross is displayed on the screen.

After rsfMRI, participants will complete a self-referential encoding task (SRET) in the scanner during the MRI localizer session [87]. In the task, participants will see either a positively or negatively-valenced word on each trial, and in separate blocks will be asked (1) if the word describes them, (2) if the word describes a friend, or (3) whether the word has a positive valence (PsychoPy code available at https://github.com/pab2163/mindful_brain_project). Participants will complete 2 runs (6:28 each, 324 volumes) of the task during collection of EPI data using a sequence identical to the rsfMRI without the VSend functor.

### Mindfulness training

All participants will be coached by a study staff member in mindfulness using a semi-structured and manualized 45-min mindfulness training protocol (manual available at https://github.com/pab2163/mindful_brain_project/tree/main/materials). The training will be administered at the in-person MRI visit with the aim of learning mental noting, a core mindfulness technique that participants will be taught to employ during real-time neurofeedback [64, 88]. Mental noting is a major component of Vipassana (insight mindfulness meditation) with key principles including concentration, observing sensory experience, not ‘efforting’, and contentment [89]. Specifically, participants will be taught to mentally label or note whatever sensation is most salient in their sensory experience from moment to moment (e.g., seeing, hearing, feeling, thinking). This practice helps individuals observe one's thoughts and feelings as temporary events in the mind, as opposed to reflections of the self that are necessarily true, which is often referred to as decentering [90]. Training also will include identifying scenarios in which mental noting could be applied in the context of each person’s life, and explaining the goal of using these strategies to manage distress across daily experiences. In addition, training will include brief psychoeducation on brain networks recruited for mindfulness versus mind-wandering, and an explanation that the MRI feedback will be based on relative activation of these networks. This brief mindfulness training session is not designed to contain all components of full-scale mindfulness treatments (e.g., Mindfulness-Based Stress Reduction Therapy) [91].

Prior to MRI Session 2, study staff will demonstrate the noting practice by verbalizing their mental labels out loud, and then ask the participant to do the same. The study staff member will observe and provide guidance, having the participant repeat the practice out loud as needed up to three times. Participants will then complete three silent practices of mental noting with distractions in the background, specifically, pre-recorded stories (see the mindfulness manual link above for detail). Participant’s success using mindfulness will be evaluated by a decrease in their ability to recall details from the story, which will be compared to their ability to recall details in a previous no-mindfulness baseline story. Finally, participants will complete a silent mental noting practice while viewing a simulation of real-time neurofeedback, including scanner sounds, on a laptop.

### Personalized brain network generation

During the Mindfulness Training, rsfMRI data from MRI Session 1 will be preprocessed using FSL 6.0 [92]. Preprocessing will include: (1) realignment of EPI volumes and calculation of head motion parameters using MCFLIRT, (2) brain extraction with BET2, (3) spatial smoothing (5 mm FWHM), and (4) high-pass filtering (0.01 Hz threshold). We note that preprocessing of rsfMRI data for personalized brain network generalization will prioritize speed for the current study, as personalized networks will need to be generated within the < 1 h interval between MRI Session 1 and MRI Session 2. Thus, some steps that would ordinarily be included in post-hoc rsfMRI preprocessing (e.g., nuisance regression, co-registration using anatomical scans, susceptibility distortion correction) will be skipped during personalized brain network generation. We will conduct subsequent analysis in native space to reduce warping of functional images.

Independent components analysis (ICA) will be performed on the preprocessed resting-state data using Melodic ICA version 3.15. Both rsfMRI runs will be used for ICA, unless fewer than 125 volumes (2.5 min) are available for either run (i.e., if the run is stopped early), in which case a single run of ≥ 125 volumes will be used. Prior to ICA, rigid body correction and brain extraction will be run for each rsfMRI run, then both rsfMRI runs will be registered to the median volume of the first run using FLIRT, and after preprocessing runs will be concatenated in time (multi-session temporal concatenation). If the runs differ in length, volumes will be removed from the end of the longer run to match the length of the shorter one. If only one resting-state run is available, no temporal concatenation will be required. To ensure a broad coverage of relevant components, 35 components will be extracted from the ICA.

**Fig. 3:**
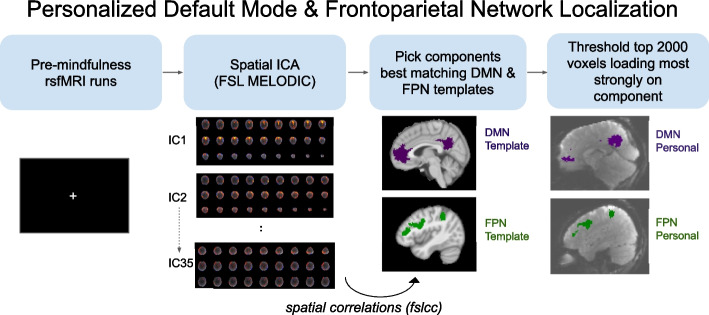
Personalized DMN and FPN Mask Generation. Examples of personalized DMN and FPN masks are shown for a pilot scan in native space

After ICA, transformations between the functional data and the MNI152 standard space (non-linear 6th generation symmetric [MNI152NLin6Sym] as used by FSL 6.0 [93]) will be calculated using FLIRT (nb: anatomical scans will not be used in this registration step). Using the inverse of this registration matrix, masks of the DMN and FPN derived from resting-state data of approximately 1000 participants (Yeo-17 DefaultA [N14], Yeo-17 ContA [N11]) [94] will be warped to the median volume of the participant’s first rsfMRI run (i.e., the native space in which the ICA was run). The DMN mask was selected to ensure that the template included mPFC and PCC, as well as angular gyrus (masks in template space can be found at https://github.com/pab2163/mindful_brain_project). FSL’s fslcc tool will be used to calculate spatial correlations between each ICA component extracted from the participant’s rsfMRI runs and the DMN and FPN masks, respectively. Components with the highest absolute value correlations with each network will be selected to obtain participant-specific DMN and FPN components. Personalized DMN and FPN masks will be created by first masking the selected ICA components to only include voxels within the template masks (warped to functional native space), thresholding each component to include the 2000 voxels loading most strongly (highest positive weights) on the respective component, then binarizing the mask (Fig. 3).

All mask generation steps will be conducted on a Linux laptop (Ubuntu) on-site after the localizer session. Piloting indicated the personalized network generation took 30–40 min for 2 runs of rsfMRI (10 min of data), and 15–20 min for 1 run (5 min of data).

### MRI session 2 (mindfulness-based real-time neurofeedback)

In MRI Session 2, a 2-volume EPI scan using identical acquisition parameters to the previous EPI runs will first be acquired for registering the personalized DMN and FPN masks to the current native (functional) space. Masks will be registered to the current functional space using FLIRT [95] and multiplied by a whole-brain mask eroded by one voxel to avoid inclusion of voxels near or off the edge of the brain. Participants will then complete two more 5-min rsfMRI runs and fieldmaps identical to those in MRI Session 1 (Pre-neurofeedback rsfMRI runs used for Primary Analyses). Next, all participants will complete a no-neurofeedback run where they will be instructed to practice mental noting without neurofeedback. Participants will either complete 5 (15-min dose condition) or 10 (30-min dose condition) runs with neurofeedback. All participants will complete a second no-neurofeedback run after the 5th neurofeedback run, and participants in the 30-min dose condition will complete a 3rd no-neurofeedback run after the 10th neurofeedback run (Fig. 2). Both no-neurofeedback and neurofeedback runs will be 3 min (150 volumes) and will be collected using identical acquisition parameters to all other EPI runs. For neurofeedback runs, the Siemens motion-corrected (MoCo Series) output will also be enabled, and images will be exported in real time to the processing laptop. After neurofeedback, participants will complete two additional 5-min rsfMRI runs (Post-neurofeedback rsfMRI runs used for Primary Analyses), followed by two more SRET runs with the same parameters noted. Each run of the SRET will use different word stimulus sets.

### Mindfulness-based real-time neurofeedback procedure

**Fig. 4:**
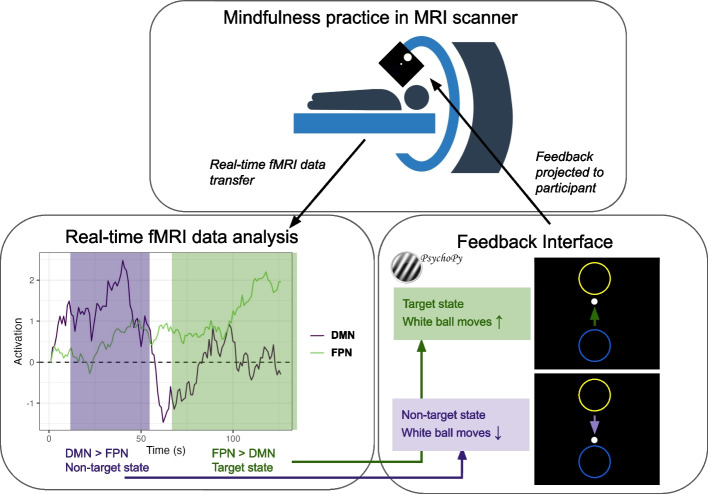
Mindfulness-based Real-time fMRI Neurofeedback Schematic. Participants will be instructed to practice mental noting while undergoing fMRI (top panel). In real time, fMRI data will be analyzed using Multivariate and Univariate Real-time Functional Imaging (MURFI) for FPN relative to DMN activation (bottom left panel). The targeted state is greater FPN relative to DMN activation. The difference between FPN and DMN activation will determine the velocity of a small white ball upwards (when FPN > DMN) and downwards (when DMN > FPN) towards two larger circles displayed to the participant via PsychoPy (bottom right panel). A free stock image from Vecteezy.com was used in this figure

During both no-neurofeedback and neurofeedback runs, participants will see a central white ball with larger circles above and below it on the screen presented via PsychoPy [96] (Fig. 4). During the no-neurofeedback runs, participants will be instructed to practice mental noting, and told that the display will not move. Before the first neurofeedback run, participants will be instructed to continue mental noting while neurofeedback based on their brains is delivered to help their practice. Participants will see instructions indicating that when the white ball moves up towards the top circle, this corresponds with the noting practice. Additionally, participants will be told not to pay too much attention to the ball, but rather, “*Try to focus mostly on the Noting Practice by being aware of your sensations from moment to moment and silently making a note in your mind. You can check the screen every once in a while to see where the ball is going*”. At the beginning of each run (both no-neurofeedback and neurofeedback runs) is a 30-s baseline displaying a fixation cross where participants will be instructed to relax and not practice mental noting. Following this, the ball and circles will appear on the screen and participants will be instructed to practice mental noting for the remaining 2.5 min of the run.

During neurofeedback runs, the velocity of the central white ball will be determined by the Positive Diametric Activity (PDA) metric, which is defined as the difference (in standard deviations) between FPN > DMN activation estimates [88]. We note that the goal of this feedback is to encourage more negative functional connectivity (anticorrelation) between the DMN and FPN, and feedback is based on activation due to methodological challenges with delivering functional connectivity feedback in real-time [97]. Accordingly, the ball will move upwards when FPN > DMN activation estimates for the last volume collected are positive and downwards if negative, at a speed proportional to the magnitude of difference. As the moment-to-moment velocity (rather than position) of the ball will be determined by the PDA metric, the ball will move smoothly and continuously, although rapid accelerations and decelerations will be possible. If the ball reaches the center of the top or bottom circle, the center of that circle will flash white for one volume to reflect a hit, then the ball will return to the center. If for a given volume either FPN or DMN activation estimates are an outlier more than 2 standard deviations away from the baseline (e.g., due to a head motion spike), the ball will pause moving until non-outlier measurements are received. Timecourses of the PDA metrics and ball position will also be recorded for post-hoc analyses.

The PDA contrast required to move the ball into the circles will be adaptively calibrated both within and between neurofeedback runs. Within runs, after a hit to either the lower or upper circle, the radius of the circle that was hit will shrink by 10%. After a run is completed, the velocity of the ball given the same PDA magnitude will increase 25% for the next run if there were < 3 hits in the previous run, and decrease by 25% if there were > 5 hits. If there were 3–5 hits, the velocity will remain the same. No monetary rewards will be awarded based on neurofeedback performance [98].

At the end of each neurofeedback run, participants will answer questions on: (1) how often they were using the noting practice (from “Never” to “Always”), (2) how often they checked the position of the ball (from “Never” to “Always”), (3) how difficult it was to apply mental noting (from “Not at all” to “Very Difficult”), and (4) how calm they feel at the current moment (from “Not at all” to “Very Calm”) on a visual analog scale from 1–9 using a button box. Full task instructions and PsychoPy code are available at https://github.com/pab2163/mindful_brain_project.

### Real-time neurofeedback operationalization

Real-time neurofeedback will be calculated using multivariate and univariate real-time functional imaging (MURFI; open source code available at https://github.com/gablab/murfi2) software on the Linux processing laptop in the control room connected to the scanner via ethernet [86]. MURFI will only be used to process data for real-time fMRI neurofeedback, and not for personalized brain network generation or post-hoc fMRI preprocessing. Only motion-corrected volumes that have been realigned to the first volume of each run using Siemens motion correction (MoCo Series) will be sent to MURFI. After the first volume of EPI data is received by this computer, DMN and FPN masks in the 2-volume run functional space will be registered to this first volume via FLIRT to ensure that the masks match the functional space of the neurofeedback run. If mask generation is not possible for a participant (for example if insufficient rsfMRI data is available), the Yeo template DMN and FPN masks will be directly registered from MNI space to the first volume of the run.

To provide neurofeedback, a separate incremental general linear model (GLM) will be fit via MURFI for each of the voxels in each mask each time a subsequent EPI volume is received. Each GLM will include all prior volumes, and will include nuisance regressors for the 6 head realignment parameters extracted from the Siemens Motion Corrected DICOM headers, as well as a linear drift regressor [99]. The six head realignment nuisance regressors will be entered into the GLM as the difference in position relative to the previous volume (relative displacement) [100]. GLMs will be fit using Gentleman’s algorithm to achieve rapid calculation. To determine each voxel’s activation over baseline at time *t*, the GLM reconstruction of the expected signal at time *t* as a function of the nuisance regressors will be subtracted from the measured voxel signal at time *t,* leaving a residual signal as the estimate of the blood-oxygen-level-dependent (BOLD) signal at time *t*. This residual will be converted to a z-score relative to the mean and standard deviation of the GLM residuals of the first 25 volumes (30 s) acquired during the baseline period. This method will result in an estimate of the activation of each voxel in the DMN and FPN masks at time *t* in units of standard deviations. Within the DMN and FPN masks, overall activation will be calculated using voxel efficiency weighting, which is determined as a weighted mean across voxels, with weights set as inversely proportional to the variance of each voxel during the 30 s baseline period [86]. Prior work has indicated that this weighting method can mitigate large signal changes due to random noise and closer convergence with offline GLMs compared to taking a mean or median across voxels [86]. Piloting indicated minimal feedback delay (< 1.2 s), such that feedback based on a given volume was delivered before the next one was acquired. However, we note that all real-time fMRI feedback is delayed roughly ~ 6-8 s due to temporal properties of human hemodynamic responses that cannot possibly be resolved via faster sampling or data processing [101]. In post-hoc analyses, we will evaluate convergence of the incremental GLM with offline methods, as well as the signal-to-noise ratio for volume-by-volume real-time neurofeedback [99].

### Follow-up assessment

At the 1-month follow-up assessment, study staff blind to the dosing condition will re-administer the CDRS-R to assess depression severity in the two weeks prior to the follow-up assessment and the C-SSRS to assess suicidal thoughts and behaviors since the last visit. A follow-up service-use interview will be administered to assess changes in treatment utilization (e.g., medication, therapy) and mindfulness practice over the course of the study. Participants also will be re-administered self-report questionnaires assessing depression and anxiety (MFQ, RCADS, PSS), rumination (RRS, PTQ), mindfulness (FFMQ, SMS), mind-wandering (MWQ), and affect (PANAS-SF). Study staff will attempt to conduct follow-up assessments for all participants randomized at the MRI Visit, whether or not they completed all MRI Visit procedures (including mbNF). Participants will receive $50 for completing the follow-up assessment.

### Adverse events

We will follow the United States Office for Human Research Protections and institutional ethics review board standards for adverse event reporting, including logging adverse events in secure documentation (REDCap), reporting serious adverse events for review to the DSMB and IRB. The MRI protocol debriefing questionnaire will be collected to assess adverse or unintended effects of the mbNF procedure, including physical or psychological discomfort during MRI scanning. Adverse events arising from participant reports and interviews, including suicidal thoughts and behaviors, will be reported as adverse events.

### Statistical analyses

Quality control checks will be conducted for data of all modalities before analysis, including inspection of missing data, examining ranges and distributions, and checking modeling assumptions. Analyses will include age (years) as a covariate. Analyses of fMRI data will also include covariates for head motion in each run (mean Jenkinson Framewise Displacement).

Analyses will be conducted using the intention-to-treat principle, such that all available data from all participants *randomized* to receive 15-min versus 30-min of mbNF will be analyzed [102]. Specifically, all available data passing quality control checks will be analyzed from participants who were randomized to a dose condition at the MRI visit, whether or not participants complete the intended duration of mbNF. Although we anticipate that the majority of participants will complete all study procedures, attrition and missing data (i.e. failing MRI quality assurance) will be reported. Imputation will be conducted at the item level for incomplete self-report measures with < 10% of items missing, though missing data for fMRI or fully missing self-report outcome measures will not be imputed.

Linear mixed-effects models using all available data will be used to test all primary hypotheses using the lme4 package [103] with the most up-to-date version of the R statistical software [104]. For all tests, we will report 95% CI and use 2-sided *p*-values with alpha level of 0.05 for significance. Robust estimation will be conducted if outliers ≥ 3 standard deviations from the mean are identified for outcome variables, and Bayesian estimation via the brms package will be used if frequentist analyses exhibit numerical problems resulting in convergence issues [105]. All models will include random intercepts, and for each analysis separately, model comparison (using the AIC metric, or PSIS-LOO for Bayesian models) will determine whether models will also include random slopes (as indicated in the model syntax below; see https://github.com/pab2163/mindful_brain_project/tree/main/materials for full notation) for each participant (Table 2).

**Table 2:** Primary and secondary analysis hypotheses, outcomes, and methods

Aim	Hypothesis	Outcome Measure	Analysis Method	Term of Interest
**Primary Analyses**				
1: Target Engagement (Analyzed as Single-Group)	Within-DMN functional connectivity will be reduced following mbNF	Resting-state functional connectivity between medial prefrontal cortex (mPFC) and posterior cingulate cortex (PCC)	Linear mixed-effects regression model	Main Effect of Time (pre > post)
2: Dosing Impact on Target Engagement (Analyzed as Parallel-Group)	Participants in the 30-min mbNF condition will show greater reductions in within-DMN functional connectivity	Resting-state functional connectivity between medial prefrontal cortex (mPFC) and posterior cingulate cortex (PCC)	Linear mixed-effects regression model	Time X Dose Interaction
**Secondary Analyses**				
Clinical Outcomes (Analyzed as Single-Group)	Depressive symptoms and rumination will be reduced following mbNF	CDRS-R, MFQ, and RRS; EMA reports of depression symptoms and rumination	Linear mixed-effects regression model	Main Effect of Time (pre > post)
Dosing Impact on Clinical Outcomes (Analyzed as Parallel-Group)	Participants in the 30-min mbNF condition will show greater clinical improvement	CDRS-R, MFQ, and RRS; EMA reports of depression symptoms and rumination	Linear mixed-effects regression model	Time X Dose Interaction

### Data preparation and preprocessing

For all post-hoc analyses, fMRI data will be preprocessed using the most up-to-date stable version of standardized analysis pipelines (fMRIPrep or HCP minimal pipeline) at the time of analysis. Current pipelines include skull-stripping, realignment, registration, normalization, susceptibility distortion correction, automated tissue segmentation, and confound regressor generation [106, 107].

Preprocessed data and confounds will be further processed for nuisance regression and functional connectivity analysis. As best practices for nuisance regression are still evolving over time, we will adhere to current standards of nuisance regression at the time of analysis. Given current recommendations, however, we anticipate including nuisance regressors for head motion (12; 3 translation, 3 rotation, and first derivatives), aCompCor (top 5 PCA components from white matter and cerebrospinal fluid [108]), and linear drift. A potentially consequential choice for nuisance regression is whether to include a regressor with the mean gray matter signal (global signal regression [GSR]), as such a “global” signal likely contains both neural and artifactual (motion, physiological) components [109, 110]. Given prior work indicating that GSR can mitigate physiological confounds in real-time neurofeedback studies [97], we will perform and report all primary analyses both with and without GSR. As part of processing, we will include a bandpass filter (current recommendations indicate 0.008-0.09 Hz) applied to fMRI data, and volumes with excessive head motion will be censored [111, 112]. FMRI data also will undergo visual quality control at several stages from raw data to preprocessed outputs [113, 114].

Functional connectivity will be calculated as the product-moment correlation between the average timecourses of primary analysis DMN regions of interest (ROIs) from rsfMRI runs co-registered to the MNI152Lin2009cAsym template (the standard fMRIPrep brain template). Primary ROIs for this study will be the mPFC and PCC voxels, respectively, within participant-specific masks used for real-time neurofeedback. In the case that any participant-specific mPFC or PCC regions contain < 200 voxels, the template mask (defined via the Yeo17 DefaultA parcellation) for that region will be used instead (https://github.com/pab2163/mindful_brain_project/tree/main/materials/ROI). Correlation coefficients will be extracted for each rsfMRI run separately, and Fisher r-to-z-transformed for group-level analysis.

### Aim 1: target engagement (analyzed as single-group)

Across doses, we will test whether decreases in within-DMN (mPFC-PCC) functional connectivity occur (during MRI Session 2) from pre- to post-mbNF. MPFC-PCC functional connectivity estimates for each rsfMRI run (including pre-mbNF runs) will be treated as the outcome variable in linear mixed-effects models, with time (0 = pre-mbNF, 1 = post-mbNF) as the focal predictor, such that there will be 4 observations (2 pre-mbNF and 2 post-mbNF runs) for each participant with complete data:

$$DMN\_connectivity \sim time + framewise\_displacement + age + (time | id)$$

### Aim 2: Dosing Impact on Target Engagement (Analyzed as parallel-group)

We will test dosing effects of mbNF (15-min versus 30-min condition) on change in mPFC-PCC connectivity (during MRI Session 2) from pre- to post-mbNF. Models will be similar to Aim 1, with an added time (0 = pre-mbNF, 1 = post-mbNF) x dose (15-min versus 30-min condition) interaction term to test differential effects of dosing on pre-post change as follows:

$$DMN\_connectivity \sim dose*time + framewise\_displacement + age + (time | id)$$

### Secondary analyses

#### Clinical outcomes (analyzed as single-group)

Across doses, analyses will explore changes in clinical outcomes from pre- to post-mbNF. Analyses will examine changes from baseline to the 1-month follow-up in depression symptoms using the self-reports (MFQ) and interviewer assessments (CDRS-R), as well as self-reported rumination using the RRS. Sum scores will be used as outcome measures for the MFQ and RRS. Further analyses will explore changes in EMA reports of depressive symptoms (sum of PHQ-2 EMA items) and rumination (sum of RRS & PTQ EMA items). Change will be examined from pre-mbNF to both the week immediately after and the week of the 1-month follow-up. Clinical outcomes will each be analyzed similarly to primary analyses using linear mixed-effects regression models as follows:

$$clinical\_measure \sim time + age + (time | id)$$

#### Dosing Impact on Clinical Outcomes (Analyzed as parallel-group)

Secondary analyses will also test dosing effects of mbNF (15-min versus 30-min condition) on change in clinical outcomes from pre- to post-mbNF as follows:

$$clinical\_measure \sim dose*time + age + (time | id)$$

Secondary analyses each involve tests of 7 outcome measures (EMA reports of depression (1) and rumination (2) the week following mbNF; EMA reports of depression (3) and rumination (4) the week of the 1-month follow-up, and CDRS-R (5), MFQ (6), and RRS brooding subscale (7) at 1-month follow-up. For EMA analyses, participants with ≥ 5 responses will be included. Given 4 tests for depression symptom outcomes and 3 tests for rumination outcomes for secondary analyses respectively, the Benjamini–Hochberg procedure will be used to control the false discovery rate at an alpha level of 0.05 [115].

### Sensitivity analyses

Analyses for Aims 1 and 2 will be conducted both with and without a global signal regression step during fMRI preprocessing. Sensitivity analyses will also be conducted including all randomization variables (i.e., site, sex assigned at birth, prior mindfulness experience, service use) as separate binary covariates [116]. Additionally, to account for potential differences in pre-mbNF DMN connectivity, Aim 2 analyses of dosing will be conducted excluding the main effect term for dose [117]. Last, sensitivity analyses will address the degree to which results are sensitive to data missing not at random (MNAR). For these analyses, missing outcome values will be assumed MNAR and iteratively replaced with values ranging from -2SD to + 2SD from the mean of respective observed values. Separate analyses for each replacement case will test the degree of robustness of results under varied MNAR scenarios [118].

### Exploratory analyses

#### Within-DMN connectivity

In addition to mPFC-PCC connectivity, we will also test connectivity change between a larger set of DMN nodes including mPFC, PCC, subgenual anterior cingulate cortex, angular gyrus, and lateral temporal cortex. We anticipate reduced functional connectivity among distributed nodes within the DMN beyond mPFC and PCC.

#### Default mode network and frontoparietal network functional connectivity

In parallel analyses to Primary Aims 1 and 2, we will test changes in functional connectivity between the DMN and FPN from pre-post mbNF, as well as dosing impacts. We anticipate stronger negative correlations (anti-correlations) between DMN-FPN post-mbNF.

#### Acceptability of mbNF

Analyses will also examine in-scanner and post-mbNF ratings of participants’ experience with mbNF to assess acceptability and adherence.

### Power calculation

G*power was used to estimate required sample sizes [119]. For Aim 1, achieving 80% power will require an estimated 76 participants (allowing ~ 15% data loss from *N* = 90) to detect significant (*p* < 0.05) within-participant change of small-medium effect sizes (f > 0.19), assuming sphericity and *r* > 0.3 between repeated measures. For Aim 2, achieving 80% power will require 38 participants in each dosing group (allowing ~ 15% data loss from *N* = 45 per group) to detect *p* < 0.05 significant dosing group x time interactions of medium effect sizes (f > 0.25), assuming sphericity and *r* > 0.3 between repeated measures.

### Data management and dissemination

At the time of submission of this manuscript, data acquisition has not yet begun. A cred-nf checklist for the study protocol can be found at https://github.com/pab2163/mindful_brain_project/tree/main/materials. Study data will be stored securely on encrypted and password-protected platforms via REDCap [120], Flywheel (https://flywheel.io/), and servers at the New York State Psychiatric Institute and Northeastern University. Any physical documents that link participant ID numbers to identifying information will be stored in a locked filing cabinet or storage unit in an area with limited access. Data will be analyzed during ongoing acquisition to ensure quality and to present for scientific audiences at conferences. Once data collection is complete, final analyses will be posted as preprints, and submitted to peer-reviewed journals and scientific conferences. Authorship will be determined using the Contributor Roles Taxonomy [121]. All de-identified data will be shared via the United States National Institute of Mental Health Data Archive (https://nda.nih.gov/), and results, including adverse event reporting, will be uploaded to clinicaltrials.gov (trial identifier: NCT05617495). Code for statistical analyses will also be shared publicly.

### Data safety monitoring board

An independent Data Safety Monitoring Board of 3 members with expertise in clinical trials, biostatistics, neuroimaging, and depression will oversee this trial. This group will meet with investigators every 6 months. No members of the Data Safety Monitoring Board are employed at Columbia University, New York State Psychiatric Institute, or Northeastern University, nor do they have current or recent collaborations with investigators or any roles in funding.

## Discussion

Novel personalized and non-invasive treatments for depression are urgently needed, especially among adolescents where MDD prevalence has been steadily increasing [2]. Targeting rumination and its neurobiological foundations may be crucial [17]. The current study will attempt to address this gap through testing whether mindfulness-based neurofeedback (mbNF) decreases functional connectivity within the DMN among depressed adolescents. In addition, this study will clarify whether a single 15-min versus 30-min dose is more effective in downregulating such DMN connectivity, and relatedly, whether a given dose leads to more optimal clinical outcomes. Follow-up research also will be conducted to explore clinical outcomes collapsed across mbNF doses and multiple metrics of real-time neurofeedback performance, as well as impacts on dynamic functional connectivity [122], self-referential processing (SRET) and sustained attention (gradCPT).

This multisite protocol will result in several key contributions to the field. First, this study will test mindfulness-based real-time fMRI neurofeedback as a potential treatment specifically for adolescents with MDD. The current protocol, to our knowledge, is one of very few studies testing any form of fMRI neurofeedback for depressed adolescents [52, 53], and thus will contribute to understanding of potential neural and clinical target mechanisms in this population. In addition, the current study will be one of the largest real-time neurofeedback samples to date, which will enhance statistical power. Finally, the current protocol also will use EMA to measure changes in mindfulness, rumination, and depression symptoms with high ecological validity and in real time [123, 124]. Our longitudinal methods measuring symptom change post-mbNF and up to a month following will benefit the characterization of courses of clinical response.

That the current study tests a single session of real-time fMRI neurofeedback is both a key strength and a limitation. MRI is expensive and constrained to geographical locations where scanners are accessible [125], and thus, single-session treatments may be more feasible for many patients. A single session of mbNF also may be helpful in conjunction with medication or psychotherapy, in particular as a “booster” for building mindfulness skills in an existing course of therapy. On the other hand, single-session real-time neurofeedback may not be as effective in achieving long-term neurobiological changes and reductions in symptoms [126]. Future studies comparing single-session and multi-session interventions on long-term outcomes will be needed to optimize mbNF schedules [67].

An additional limitation of the present study, particularly for aim 1, is the absence of a true control group. The present study will not be able to fully rule out the influence of confounding factors on changes in DMN connectivity, such as placebo effects [127], changes in respiratory or cardiac physiology [97, 128], and head motion [110], as well as reductions in arousal, stress, or anxiety driven by acclimation to the study and scanner environment over the course of the session [129]. Although prior work has found that only mbNF, and not neurofeedback based on a somatomotor control region, resulted in decreased DMN connectivity [88], these confounds could impact both the neurofeedback signal itself and the rsfMRI data used for primary analysis. However, sensitivity analysis will be run to address whether effects persist after adjusting for these factors. Further, as is often the case with single-group trials without a control group, regression to the mean and placebo effects may also drive estimates of change in clinical symptoms assessed in secondary analyses [130]. Yet, any results showing an absence of change in clinical symptoms or iatrogenic effects may be meaningful, as regression to the mean and placebo effects typically inflate estimates of symptom improvement. Further, such confounding mechanisms are not expected to explain differences in change between participants randomized to 15-min versus 30-min mbNF doses. Additionally, there is not yet consensus on whether decreasing functional connectivity within the DMN drives reductions in either depression symptoms or rumination [61, 131, 132]. Future work can better address mechanisms of change through randomized and double-blinded comparisons with an active control or yoked sham feedback condition [133].

In summary, this project will test whether a non-invasive personalized mbNF protocol can guide adolescents with MDD to downregulate default mode network connectivity. This protocol builds on encouraging pilot findings [64] and tests a crucial step in determining the efficacy of mbNF for regulating neurobiology related to depressogenic rumination. This work aims to bridge gaps between cognitive and affective neuroscience and current treatment methods to inform precision-based interventions for improving clinical outcomes among adolescents with MDD.

## Data Availability

All materials are publicly available at https://github.com/pab2163/mindful_brain_project. Code and de-identified study data will also be made publicly available (see the Data Management and Dissemination section for details).

## Abbreviations

BOLD: Blood-oxygen-level-dependent
C-SSRS: Columbia-Suicide Severity Rating Scale
CDRS-R: Children’s Depression Rating Scale-Revised
CU: Columbia University
CUIMC: Columbia University Irving Medical Center
DMN: Default mode network
EMA: Ecological momentary assessment
EPI: Echo-planar imaging
FA: Flip angle
FFMQ: Five-Facet Mindfulness Questionnaire
fMRI: Functional magnetic resonance imaging
FOV: Field-of-view
FPN: Frontoparietal network
FSIQ-2: Full-Scale Intelligence Quotient-2
GAD-2: Generalized Anxiety Disorder-2 item
GLM: General linear model
gradCPT: Gradual onset continuous performance task
HIPAA: Health Insurance Portability and Accountability Act of 1996
ICA: Independent components analysis
K-SADS: Kiddie-Schedule for Affective Disorders and Schizophrenia
mbNF: Mindfulness-based real-time fMRI neurofeedback
MDD: Major Depressive Disorder
MFQ: Mood and Feelings Questionnaire
mPFC: Medial prefrontal cortex
MRI: Magnetic resonance imaging
MTL: Medial temporal lobe
MNAR: Missing not at random
MURFI: Multivariate and Univariate Real-time Functional Imaging
MWQ: Mind-Wandering Questionnaire
NEU: Northeastern University
NYSPI: New York State Psychiatric Institute
PANAS-SF: Positive and Negative Affect Schedule-Short Form
PCC: Posterior cingulate cortex
PDA: Positive Diametric Activity
PHQ-2: Patient Health Questionnaire-2 item
PSS: Perceived Stress Scale
PTQ: Perseverative Thinking Questionnaire
RCADS: Revised Children’s Anxiety and Depression Scale
RCT: Randomized clinical trial
ROI: Region of interest
rsfMRI: Resting-state functional magnetic resonance imaging
RSS: Ruminative Response Scale
sgACC: Subgenual anterior cingulate cortex
SMS: State Mindfulness Scale
SN: Salience network
SRET: Self-referential encoding task
TE: Echo time
TR: Repetition time
WASI-II: Wechsler Abbreviated Scale of Intelligence-II
